# Long-term *in situ* straw returning increased soil aggregation and aggregate associated organic carbon fractions in a paddy soil

**DOI:** 10.1016/j.heliyon.2024.e32392

**Published:** 2024-06-04

**Authors:** Jiaren Liu, Xuehai Wang, Hu Zhang, Yifei Lu, Yusef Kianpoor Kalkhajeh, Hongxiang Hu, Jieying Huang

**Affiliations:** a Anhui Province Key Lab of Farmland Ecological Conservation and Pollution Prevention, School of Resources and Environment, Anhui Agricultural University, Hefei, 230036, China; b Department of Environmental Science, College of Science, Mathematics and Technology, Wenzhou-Kean University, 88 Daxue Road, 325060, Ouhai, Wenzhou, Zhejiang Province, China

**Keywords:** Straw returning, Dissolved organic carbon, Easily oxidative organic carbon, Resistant organic carbon, Aggregation size composition

## Abstract

Despite the well-documentation of the effects of straw returning on soil structural stability and fertility, its long-term *in situ* impacts on profile aggregate size composition and organic carbon (OC) fractions remain poorly investigated. To address this research gap, the present nine-year field trial explored the co-effects of straw returning and chemical fertilization on soil total OC (TOC), dissolved OC (DOC), resistant OC (ROC), easily oxidative OC (EOC), as well as soil aggregate size composition of different soil depths (0–15, 15–30, and 30–50 cm) in a paddy field, East China. To do so, four different treatments were set up, including no straw returning plus no fertilization (CK), conventional fertilization (F), straw returning plus conventional fertilization (SF), and straw returning plus 80 % conventional fertilization (SDF). Our findings revealed that the >2 mm aggregates were dominant in all treatments, particularly in SF and SDF 0–30 cm soil layers ranging from 62 to 70 % (P < 0.05). The highest TOC contents happened in SF topsoil 0.25–2 mm aggregates (0–30 cm; 21.4 g/kg), 44.4 and 21.1 % higher than the CK and F treatments, respectively (P < 0.05). Regardless of soil depth, the highest EOC contents occurred in SDF 0.25–2 mm aggregates varying from 2.36 ± 0.1 to 7.7 ± 0.57 g/kg (P < 0.05). Further, the highest ROC and DOC contents took place in SF 0.25–2 mm and SF > 2 mm aggregates, respectively, differing from 3.86 to 15.8 g/kg and 250–413 mg/kg, respectively (P < 0.05). It is also worth noting that SF had the highest crop productivity with the seasonal yields of 3.51 and 13.5 t ha^−1^ for rapeseed and rice, respectively (P < 0.05). Altogether, our findings suggested that long-term straw returning coupled with conventional (SF) or 80 % conventional (SDF) fertilization are the most efficient schemes for the formation/stability of soil aggregates, as well as for the accumulation of different soil OC fractions and crop productivity in the Chaohu Lake agricultural soils of East China.

## 1 Introduction

Crop straw is the main agricultural byproduct, with an annual production of about 1 billion tons in China [1]. Straw residues are carbon (C) rich substances, containing enormous amounts of cellulose and hemicellulose, as well as nutrients [2,3].

Numerous studies have investigated the agricultural and environmental implications of straw returning [[4], [5], [6]]. As such, straw returning had inhibiting/decreasing effects on nitrogen (N) and phosphorus (P) leaching [7,8]. Several workers revealed that straw returning can improve soil microbial community/functionality, soil fertility/quality, and crop productivity [4,6,9]. Although straw addition might increase the emissions of N_2_O and CH_4_ from the agricultural soils [5,10].

Soil aggregates and organic C are the two key indicators for the assessment of soil fertility and quality/health [11]. Lin et al. [12] noted that soil aggregation and soil organic C fixation are the mutually reinforcing processes. Soil organic C is a major cementation material that holds soil particles together to form soil aggregates [13,14]. On the other hand, soil aggregates are the main storage sites protecting organic C against microbial mineralization/decomposition [15,16]. In addition, the rate and the size distribution of soil aggregates affect the physical stability of soil structure. The latter determines soil erodibility and fertility [17,18].

Several studies have explored the effects of straw retuning on soil organic C fractions/contents [19,20]. For instance, long-term straw returning improved the accumulations of soil active organic C, microbial biomass C, and dissolved organic C in a dryland maize [20]; and total and easily oxidative organic C contents in a double-cropped rice paddies [19]. Likewise, long-term straw returning (7 years) increased the proportions of both large (>2000 μm) and small (250–2000 μm) macro-aggregates in an Udic Haplustalf topsoil [21]. Consistently, significant increases happened in the North China Plain >2 mm soil aggregates amended with both wheat and maize straws [13].

To date, most scholars have mainly documented the effects of *in situ* straw returning on soil aggregates and aggregate-associated organic C fractions in the plough layer. Nevertheless, this issue has remained poorly investigated in subsoil layers, especially coupled with chemical fertilization.

Paddy soils include a relatively shallow cultivation layer, with a poorly permissible thick and solid bottom. Thus, straw returning to the plough layer of paddy soils might be an appropriate practice to reduce the bulk density and to improve the porosity, facilitating the transformation/migration of soil solution and its solutes from topsoil to the deeper soil layers via subsurface leaching [11,13].

Altogether, the present work was primarily aimed to explore the effects of co-application of straw and chemical fertilizer on soil profile aggregate size composition/distribution and their associated organic C fractions in a paddy field (0–50 cm), Chaohu Lake Region, East China, subjected to a nine-year *in situ* straw returning. Furthermore, we assessed the annual yields of rice and rapeseed in straw amended soils compared with non-straw amended and chemically fertilized soils. Finally, stakeholders and decision makers are hoped to apply the results of this study as a practical basis to improve the soil C pool, soil fertility, and crop productivity both at the regional and global scales.

## 2 Materials and methods

### 2.1 Study area

The experimental base of Anhui Agricultural University, Chaohu Lake Region, Anhui Province (117°41′37″E, 31°39′37″N), East China, was selected for this study. This region belongs to the northern subtropical humid monsoon climate zone, with a mean annual temperature and precipitation of 17 °C and 1150 mm, respectively. Of total annual rainfall, about 55 % happens in May–August. The major soil type of the study area is Gleyic Stagnic Anthrosols [22], with pre-experimental topsoil (June 2013; 0–20 cm) pH of 6.1, total N of 1.37 g/kg, total P of 0.35 g/kg, total K of 8.57 g/kg, available N of 142 mg/kg, available P of 12.3 mg/kg, available K of 109 mg/kg, and total organic C of 14 g/kg.

### 2.2 Experimental design

We established the field trial in June 2013 via rice-rapeseed rotation. Herein, the major rice variety was "Gangyou 735″, planted in mid-June and harvested in early October; and the rapeseed variety was "Qin You No.10″, planted in mid-November and harvested in May of the following year.

We established four different treatments in this study: no straw returning with no fertilization (CK), conventional fertilization (F), straw returning plus conventional fertilization (SF), and straw returning plus 80 % conventional fertilization (SDF). The conventional fertilization is equivalent to chemical fertilization and it refers to the rate and the method of fertilization applied by the local farmers, mainly via compound fertilizer (N–P–K: 18-10-18) and urea (46.4 % N content). Cai et al. [23] revealed that a 20 % reduction in chemical fertilization brought about higher yields in the experimental site. Based upon which, 80 % conventional fertilization was considered as the optimized/adequate fertilization scheme via reducing the rate of chemical fertilization. All treatments were carried out in three replicates, with a total of 12 × 30 m^2^ plots (5 m × 6 m) in a randomized complete block design. Table 1 summarizes the details of the fertilization schemes in different treatments under both rapeseed and rice cultivations.

**Table 1:** Fertilization scheme for different treatments.

Crop type	Treatment	Straw C input (kg ha^−1^)	N (kg ha^−1^)		P_2_O_5_	K_2_O	Straw returning ratio (%)
			Base fertilizer	Topdressing	kg ha^−1^		
Rapeseed	CK	0	0	0	0	0	0
	F	0	196	139	109	196	0
	SF	3590	196	139	109	196	100
	SDF	3590	157	110	87	157	100
Rice	CK	0	0	0	0	0	0
	F	0	205	97	114	205	0
	SF	3880	205	97	114	205	100
	SDF	3880	165	78	90	165	100

CK: Control; F: Conventional fertilization; SF: Straw returning plus conventional fertilization; SDF: Straw returning plus 80 % conventional fertilization; N: Nitrogen.

After each harvest, the crop residues (10 cm of aboveground) were cut and returned to the depth of 5–10 cm using a harvester. The rate of straw returning was equivalent to the amount of harvested residues from the previous plantation. Please see Table 1 for more details.

### 2.3 Soil sample collection

After nine years of straw returning, soil samples were collected following the harvest of rapeseed in May 2022. Overall, a total of 12 composite soil samples were collected from the experimental site. At each experimental block, a representative soil sample was collected comprising five subsamples from three different depths (0–15, 15–30, and 30–50 cm). Collected soil samples were crashed (<10 μm), air-dried, and the impurities including the stones and crop residues were carefully removed.

### 2.4 Aggregate size composition

We applied the wet screening method to determine the aggregate size distributions [24]. In brief, 50 g of air-dried soil was soaked with deionized water in a bucket (10 cm radius × 32 cm height) for 10 min with three different sieves (0.053, 0.25, and 2 mm), placing from the top to the bottom, respectively. In accordance, 2 mm represents >2 mm aggregates; 0.25 mm refers to the 0.25–2 mm aggregates, 0.053 mm accounts for 0.053–0.25 mm aggregates, and the remaining suspended particles in the bucket were <0.053 mm aggregates. The samples were then placed into an aggregate screening instrument, and shaken vertically for 30 min at 30 r/min. Finally, the aggregates remaining on 0.053, 0.25, and 2 mm sieves were thoroughly washed using deionized water and oven-dried at 105 °C to determine their weights and percentages/ratios. The buckets were then left for an additional 48 h to collect and determine <0.053 mm aggregates.

### 2.5 Soil organic carbon fractionation

Total organic carbon (TOC) was determined via the potassium dichromate digestion method [25]. For dissolved organic C (DOC) determination, soil aggregate samples (10 g) were shaken for 1 h at an aggregate: water ratio of 1:5 (g: mL) at room temperature (25 °C; 250 r/min). Then after, the suspensions were centrifuged at 5000 r/min for 10 min, and filtered using hydrophobic 0.45 μm PTFE filter membrane (Jena Analytical Instruments (Beijing) Co., Ltd). The DOC concentrations in soil solutions were measured via an automatic carbon analyzer (Jena, Germany Multi N/C 3100 TOC Total Organic Carbon) [26]. Acid hydrolysis method was employed to determine the contents of resistant organic C (ROC) [27]. In accordance, 2 g soil aggregate was digested with 6 N HCl at an aggregate: 6 N HCl acid ratio of 1: 2.5 at 115 °C for 16 h. Solutions were gently shaken by hand every 1 h. After cooling, the residues were washed three times with distilled water to remove chlorine. Afterwards, the residues were oven dried at 55 °C and sieved (0.149 mm) to determine ROC contents via potassium dichromate external digestion method. To determine EOC, 2 g soil aggregate was shaken with 25 mL 333 mmol L^−1^ KMnO_4_ in 50 mL centrifuge tubes for 1 h at 250 r/min. Then, the mixture was centrifuged at 3500r/min for 5 min, diluted with deionized water at 1: 250, and the absorbance of the diluted solution was measured at 565 nm [28]. All the measurements were carried out in three replicates.

### 2.6 Data analysis

The basic statistical analyses were carried out using Microsoft Excel 2016 on the raw data. The effects of straw returning on profile distribution of aggregates’ associated organic C fractions among the different treatments and within the treatments were tested via one-way ANOVA at a significance level of 0.05 in SPSS 22 (IBM Corporation, NY, USA). All graphs were performed in Origin 9 (OriginLab Inc, Northampton, MA).

## 3 Results

### 3.1 Soil aggregate size composition

**Fig. 1:**
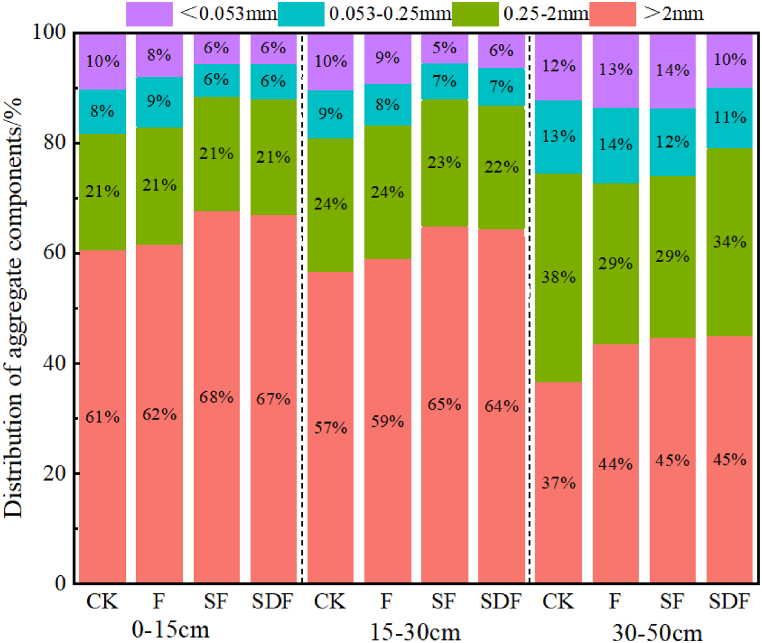
Profile distribution of different soil aggregates under various treatments. CK: Control; F: Conventional fertilization: SF: Straw returning plus conventional fertilization; SDF: Straw returning plus 80 % conventional fertilization.

Fig. 1 illustrates the profile distribution of different soil aggregates under various treatments. Overall, the > 2 mm aggregates were dominant in all soil layers, except at 30–50 cm for CK, although a gradual reduction happened in their proportions from 0 to 15 cm (59.7–70.8 %) to 30–50 cm (35.5–51.3 %). In 0–30 cm soil layers, the > 2 mm aggregates were significantly higher in SF and SDF treatments than in F treatment by 2.37–13.2 % (P < 0.05); no significant differences happened in the proportions of 0.25–2 mm aggregates among the different treatments. Unlike, for all treatments, the highest contents of 0.25–2 mm aggregates appeared in 30–50 cm soil layers ranging from 22.8 to 41 %. This suggests a reduction in the size of aggregates with increasing the soil depth (P < 0.05). Likewise, the highest proportions of 0.053–0.25 mm aggregates happened in the CK and F (30–50 cm) treatments, ranging from 10.7 to 15.6 %. It is also worth noting that the highest proportions of < 0.053 mm aggregates took place in F and SF (30–50 cm) treatments, ranging from 12.3 to 16.8 %.

### 3.2 Aggregate-associated total organic carbon

**Fig. 2:**
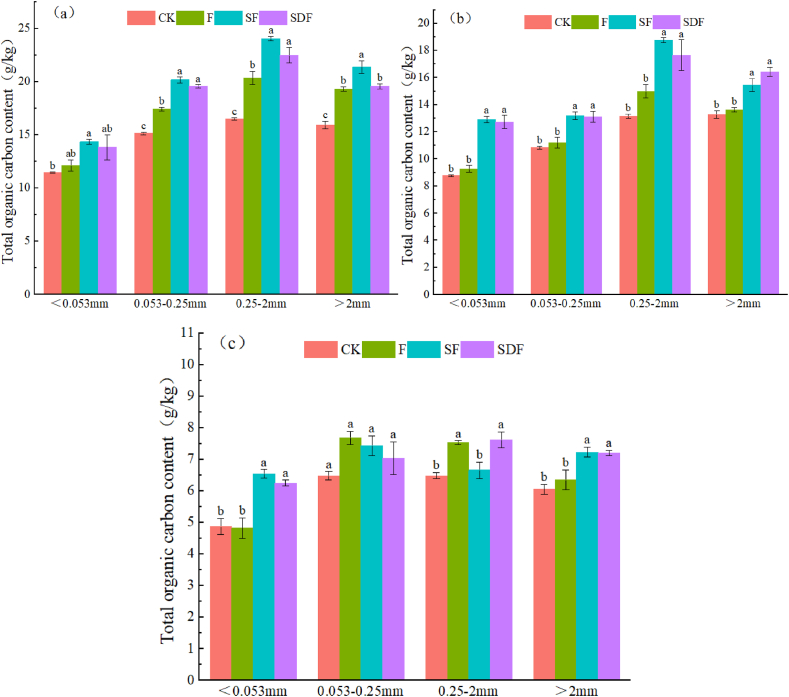
Profile distribution of total organic carbon (TOC) of different soil aggregates under various treatments; a. b, and c represent the TOC contents of soil aggregates in 0–15, 15–30, and 30–50 cm soil layers, respectively. Each bar is the standard error of three replicates. CK: Control; F: Conventional fertilization: SF: Straw returning plus conventional fertilization; SDF: Straw returning plus 80 % conventional fertilization. Columns followed by different lowercase letters are signiﬁcantly different at P < 0.05.

The profile contents of total organic carbon (TOC) of different soil aggregates under the different treatments are shown in Fig. 2 (a – c). Overall, for all treatments, reductions occurred in the aggregates’ TOC contents with soil depth (P < 0.05). In accordance, the mean TOC contents of 0–15, 15–30, and 30–50 cm aggregates were 17.7 ± 3.7, 13.57 ± 2.76, and 6.64 7 ± 0.92 g/kg, respectively (P < 0.05) [Fig. 2 (a – c)]. Regardless of soil depth, the highest TOC contents appeared in 0.25–2 mm aggregates ranging from 6.2 to 24.3 g/kg. In particular, the highest TOC contents happened in SF amended topsoil (0–30 cm) 0.25–2 mm aggregates, with a mean of 21.4 ± 2.9 (P < 0.05) [Fig. 2 (a and b)]. Fig. 2(a) shows that, in 0–15 cm soil layers, the mean TOC contents of F (17.3 g/kg), SF (20 g/kg), and SDF (18.7 g/kg) aggregates were significantly higher than the CK aggregates (14.7 g/kg) (P < 0.05). In 15–30 cm, the mean TOC contents of both SF and SDF aggregates were significantly over 22 and 30 % higher than the F and CK aggregates, respectively (P < 0.05) [Fig. 2(b)]. However, in 30–50 cm, on one side, the TOC contents of < 0.053 and > 2 mm soil aggregates had significant differences between SF (6.53 and 7.23 g/kg) and SDF (6.25 and 7.2 g/kg); and between F (4.82 and 6.35 g/kg) and CK (4.86 and 6.05 g/kg) on the other side (P < 0.05) [Fig. 2(c)].

### 3.3 Aggregate-associated easily oxidative organic carbon

Table 2 summarizes the contents of profile EOC of different soil aggregates under different treatments. Regardless of soil depth and aggregate size, the highest EOC contents happened in SDF aggregates, with a significant reduction with soil depth particularly for 30–50 cm (P < 0.05). In accordance, the mean EOC content of 0–15 cm SDF aggregates (6.58 g/kg) was 6.56, 11.1, and 23 % higher than the SF, F, and CK treatments, respectively. These observations were rather double and triple for F and CK, respectively, in 15–30 cm; and over two times, four times, and five times for SF, F, and CK, respectively, in 30–50 cm (Table 2). Similar to TOC, in all treatments, the highest contents of EOC appeared in 0.25–2 mm aggregates, followed by > 2, 0.053–0.25, and < 0.053 mm soil aggregates, respectively. For instance, the mean EOC content of 0.25–2 mm aggregates of all treatments (6.85 g/kg) was 7.28, 15.2, and 47 % higher than > 2, 0.053–0.25, and < 0.053 mm aggregates, respectively (Table 2).

**Table 2:** Contents of easily oxidative organic carbon of aggregates in 0–50 cm soil layers under different treatments (g/kg).

Soil layer (cm)	Treatment	Soil aggregates			
		＜ 0.053 mm	0.053–0.25 mm	0.25–2 mm	>2 mm
0–15	CK	3.76 ± 0.12c	4.97 ± 0.36b	5.93 ± 0.54b	5.15 ± 0.14b
	F	4.35 ± 0.29BCE	6.04 ± 0.44a	6.73 ± 0.04 ab	6.56 ± 0.26a
	SF	4.60 ± 0.13 ab	6.27 ± 0.49a	7.04 ± 0.25 ab	6.78 ± 0.56a
	SDF	5.03 ± 0.18a	6.51 ± 0.07a	7.71 ± 0.33a	7.06 ± 0.13a
15–30	CK	3.31 ± 0.08c	3.55 ± 0.14c	4.51 ± 0.16d	3.60 ± 0.29c
	F	4.03 ± 0.07b	4.30 ± 0.05b	5.10 ± 0.51c	4.25 ± 0.98b
	SF	4.43 ± 0.16a	4.81 ± 0.86a	5.81 ± 0.43b	5.24 ± 0.52a
	SDF	4.68 ± 0.10a	5.09 ± 0.57a	6.17 ± 0.98a	5.39 ± 0.15a
30–50	CK	0.16 ± 0.04b	0.80 ± 0.08c	1.25 ± 0.07c	1.28 ± 0.24c
	F	0.24 ± 0.01b	1.42 ± 0.03b	1.78 ± 0.03b	1.47 ± 0.05BCE
	SF	0.52 ± 0.10a	1.52 ± 0.11 ab	2.16 ± 0.08a	1.84 ± 0.08 ab
	SDF	0.67 ± 0.07a	1.76 ± 0.09a	2.36 ± 0.06a	2.18 ± 0.11a

CK: Control; F: Conventional fertilization: SF: Straw returning plus conventional fertilization; SDF: Straw returning plus 80 % conventional fertilization. Each value is the mean ± SD of three replicates.

Numbers followed by different lowercase letters in the same column are signiﬁcantly different at P < 0.05.

### 3.4 Aggregate-associated resistant organic carbon

The profile contents of ROC of different soil aggregates under various treatments are presented in Table 3. Overall, for all soil aggregates, reductions happened in the contents of ROC with soil depth. Correspondingly, the mean ROC contents of 0–15, 15–30, and 30–50 cm soil aggregates were 10.7, 8.2, and 4.96 g/kg. The 0.25–2 and < 0.053 mm soil aggregates had the highest and the lowest contents of ROC, particularly in 30–50 cm soil layers (P < 0.05). For instance, regardless of the treatment, the mean ROC content of 0.25–2 mm soil aggregates (13.3 g/kg) in 0–15 cm was 75, 23.1, and 20.6 % higher than the corresponding < 0.053, 0.053–0.25, and > 2 mm aggregates, respectively (Table 3). Regardless of soil depth, the highest ROC contents happened in SF aggregates, except for 0.25–2 mm aggregates in 30–50 cm, particularly compared with the CK (P < 0.05). In accordance, in 0–15 cm soil layer, the mean ROC content of SF (12.4 g/kg) was 50.6, 16.2, and 7.98 % higher than the CK, F, and SDF, respectively, corresponding to 27.9, 16.8, and 0.2 %, respectively, in 15–30 cm soil layer (Table 3).

**Table 3:** Resistant organic carbon (ROC) contents of aggregates in 0–50 cm soil layers under different treatments (g/kg).

Soil layer (cm)	Treatment	Soil aggregates			
		＜ 0.053 mm	0.053–0.25 mm	0.25–2 mm	＞ 2 mm
0–15	CK	6.53 ± 0.70b	8.11 ± 0.38c	9.71 ± 0.26b	8.61 ± 0.32c
	F	7.42 ± 0.08b	10.6 ± 0.14b	14.1 ± 0.25a	10.6 ± 0.42b
	SF	8.84 ± 0.33a	12.6 ± 0.74 ab	15.1 ± 0.40a	13.1 ± 0.44a
	SDF	7.67 ± 0.18 ab	12.00 ± 0.26a	14.4 ± 0.14a	11.9 ± 0.29 ab
15–30	CK	5.04 ± 0.44b	6.54 ± 0.13c	8.82 ± 0.69b	7.83 ± 0.06b
	F	5.72 ± 0.35b	7.02 ± 0.10b	9.23 ± 0.15 ab	8.94 ± 0.99 ab
	SF	7.60 ± 0.37a	7.56 ± 0.16a	11.6 ± 1.20a	9.35 ± 0.23 ab
	SDF	7.14 ± 0.19a	7.40 ± 0.12a	10.9 ± 0.08 ab	10.6 ± 0.20a
30–50	CK	4.55 ± 0.18a	5.09 ± 0.27a	4.83 ± 0.40 ab	4.14 ± 0.06b
	F	4.85 ± 0.11a	5.19 ± 0.46a	5.56 ± 0.43a	4.77 ± 0.13 ab
	SF	5.01 ± 0.16a	5.46 ± 0.41a	5.03 ± 0.24b	5.18 ± 0.31a
	SDF	4.82 ± 0.08a	5.25 ± 0.46a	5.1 ± 0.23 ab	4.58 ± 0.14 ab

CK: Control; F: Conventional fertilization: SF: Straw returning plus conventional fertilization; SDF: Straw returning plus 80 % conventional fertilization. Each value is the mean ± SD of three replicates.

Numbers followed by different lowercase letters in the same column are signiﬁcantly different at P < 0.05.

### 3.5 Aggregate-associated dissolved organic carbon

Table 4 presents the contents of aggregates’-associated DOC of different soil layers under various treatments. Reductions took place in DOC contents versus soil depth, with the mean contents of 369, 339, and 325 mg/kg for 0–15, 15–30, and 30–50 cm, respectively (Table 4). Interestingly, the DOC contents increased with increasing the size of aggregates. Hence, in all soil layers, the highest and the lowest DOC contents occurred in > 2 and < 0.053 mm aggregates, respectively (P < 0.05). For instance, regardless of treatment, the DOC contents of > 2 and < 0.053 mm aggregates were 473 and 295 mg/kg, respectively, in 0–15 cm soil layers, corresponding to 430 and 274 mg/kg, respectively, in 15–30 cm, and 411 and 158 mg/kg, respectively, in 30–50 cm soil layers (P < 0.05) (Table 4). Similar to ROC, the highest DOC contents happened in SF treatments in all soil layers (P < 0.05). In accordance, the mean aggregate associated DOC contents in SF 0–15 (449 mg/kg), 15–30 (409 mg/kg), and 30–50 cm (391 mg/kg) soil layers were over 50, 30, and 8 % higher than the corresponding CK, F, and SDF treatments, respectively (P < 0.05) (Table 4).

**Table 4:** Dissolved organic carbon (DOC) contents of aggregates in 0–50 cm soil layers under different treatments (mg/kg).

Soil layer (cm)	Treatment	Soil aggregates			
		＜ 0.053 mm	053–0.25 mm	0.25–2 mm	>2 mm
0–15	CK	242 ± 4.41b	260 ± 5.77d	301 ± 9.98d	382 ± 6.95c
	F	256 ± 5.75b	304 ± 3.03c	344 ± 4.21c	394 ± 11.2c
	SF	351 ± 3.90a	411 ± 5.66a	434 ± 11.6a	600 ± 6.55a
	SDF	330 ± 12.3a	379 ± 4.60b	403 ± 4.92b	516 ± 8.85b
15–30	CK	218 ± 5.58d	233 ± 9.28d	268 ± 11.7c	353 ± 6.01c
	F	247 ± 3.42c	281 ± 6.94c	316 ± 6.43b	381 ± 8.89c
	SF	329 ± 5.17a	388 ± 5.24a	401 ± 6.79a	517 ± 10.2a
	SDF	301 ± 1.76b	356 ± 2.89b	374 ± 4.91a	470 ± 10.4b
30–50	CK	205 ± 3.48d	221 ± 1.88d	264 ± 3.40d	342 ± 4.23d
	F	231 ± 6.36c	272 ± 5.35c	305 ± 5.25c	365 ± 1.59c
	SF	310 ± 2.85a	372 ± 3.12a	392 ± 11.5a	490 ± 9.62a
	SDF	288 ± 3.24b	339 ± 6.50b	362 ± 2.96b	447 ± 6.85b

CK: Control; F: Conventional fertilization: SF: Straw returning plus conventional fertilization; SDF: Straw returning plus 80 % conventional fertilization. Each value is the mean ± SD of three replicates.

Numbers followed by different lowercase letters in the same column are signiﬁcantly different at P < 0.05.

### 3.6 Crop yield

Table 5 shows the effects of different treatments on the annual yields of rice and rapeseed in 2022. Apparently, for both crops, the highest yields appeared in SF treatment, significantly higher than the F and CK treatments (P < 0.05). In accordance, the SF seasonal rapeseed production (3.51 t ha^−1^) was 166, 12.5, and 11.4 % higher than those of CK, F, and SDF treatments, respectively (P < 0.05) (Table 5). Consistently, the SF seasonal rice yield (13.5 t ha^−1^) was 50.7, 16.4, and 15.4 % higher than the CK, F, and SDF treatments, respectively (P < 0.05) (Table 5). It is also worth noting that no significant differences happened in the seasonal and annual productions of both crops in F and SDF treatments (Table 5).

**Table 5:** Effects of different treatments on crop yield (t ha^−1^).

Treatment	Rapeseed yield	Rice yield	Annual production
CK	1.32 ± 0.03c	8.96 ± 0.005c	10.3 ± 0.40c
F	3.12 ± 0.01b	11.6 ± 0.30b	14.7 ± 0.35b
SF	3.51 ± 0.003a	13.5 ± 0.36a	17 ± 0.41a
SDF	3.15 ± 0.09b	11.7 ± 0.26b	14.9 ± 0.24b

CK: Control; F: Conventional fertilization: SF: Straw returning plus conventional fertilization; SDF: Straw returning plus 80 % conventional fertilization. Each value is the mean ± SD of three replicates.

Numbers followed by different lowercase letters in the same column are signiﬁcantly different at P < 0.05.

## 4 Discussion

### 4.1 Soil aggregate size composition

Our findings revealed that macro-aggregates (> 2 mm) were dominant in all soil layers. In accordance, a significant increase of approximately ∼11 % appeared in the contents of soil macro-aggregates via straw returning coupled with the conventional (SF) and or 80 % conventional fertilization (SDF) compared with the F treatment [29]. This is consistent with the results of Zhao et al. [21], suggesting a higher structural/aggregate stability in the study straw amended soils [13]. However, in an intensively cultivated grey desert soil in northwestern China, long-term applications of crop residues/manure (24-year period) brought about a higher increase of 14–24 % in the contents of macro-aggregates than in this study [30]. Higher contents of >2 mm aggregates in SF and SDF treatments than the F treatment might partly attribute to the neutralizing effect of straw on soil acidification in response to the long-term chemical fertilization [31]. Hence, a higher reduction may have happened in the soil pH of F treatment, increasing the loss of exchangeable base cations that are essential for soil aggregation and structural stability [32]. In particular, higher proportions of > 2 mm aggregates in the upper soil layers (0–30 cm) might attribute to their higher accumulation of organic C fractions than in 30–50 cm layers due to the shallow straw returning in this study [Fig. 2 (a–c); Table 2, Table 3, Table 4] [13,30,33]. It is also worth noting that *in situ* straw returning provides fresh organic substrate for soil microorganisms [34]. This can improve the microbial decomposition of soil organic matter, that in turn accompanies by large productions of polysaccharide metabolites and humic substances [14,29]. The latter contribute to the formation of larger and more stable aggregates [35]. The high content of lignin in straw structure acts as a cementation core to bind the small soil particles into the large macro-aggregates [36]. Furthermore, straw returning can decrease the degrading effect of precipitation on soil aggregates/structural stability, with a subsequent reduction in leaching [37]. Contrary to our findings, a three-year field trial revealed that straw returning had no impact on soil aggregation in a cold semi-arid region [38]. Herein, an increase of over 60 % happened in >2 mm aggregates in non-straw amended soils. Nevertheless, non-significant changes or less accumulations of the smaller aggregates (< 2 mm) in our study soils were in accord with the findings of Soon and Lupwayi [38]. This might attribute to the less accumulation of organic C in these aggregates (Table 2, Table 3, Table 4) [37].

### 4.2 Aggregate-associated organic carbon fractions

Overall, in all treatments, reductions happened in the contents of organic C fractions of the different aggregates with soil depth [Fig. 2 (a–c); Table 2, Table 3, Table 4] [39]. This might attribute to the shallow straw incorporation (5–10 cm) and less plant root residues/development in the deeper soil layers, particularly within 30–50 cm. In addition, higher contents of organic C in top soil layers might attribute to the higher activity of soil microorganisms, that in turn contribute to the straw decomposition and C cycle [29,34]. Higher accumulation of organic C fractions in the upper SF and SDF soil layers might also attribute to their higher crop yield, with larger production of root exudates [39,40].

Our results suggested a mutual relationship between the formation/accumulation of aggregates and the abundance of the different organic C fractions. In particular, straw C input stimulated the formation of macro-aggregates via binding the smaller soil particles [41,42]. In accordance, total organic C fractions had higher accumulation/storage in >2 and 0.25–2 mm aggregates [13,15]. Similar observation took place in Urumqi grey desert soils [34]. This is predominantly associated to the straw C input [Table 1; Fig. 2 (a–c)] [19]. Nonetheless, chemical fertilizers supply N for soil microorganisms, accelerating the microbial straw decomposition particularly under anaerobic rice cultivation [42]. This explains higher crop biomass/TOC accumulation in SF and SDF treatments than in F and CK treatments [Fig. 2(a–c); Table 5] [4].

Higher EOC contents in 0–30 cm straw amended soils might be due to the higher microbial activity and tillage driven permeability at these layers (Table 2) [44]. Conversely, Hu et al. [45] indicated that straw returning had an insignificant effect on EOC contents due to its high C/N ratio or incomplete degradation. Others argued the losses of soil OC via the emission of greenhouse gases particularly during the anaerobic rice season [46,47]. This can be witnessed by the less EOC contents in smaller aggregates, that in turn causes less protection against OC oxidation/mineralization (Table 2). Finally, the significant reductions in the EOC contents of subsoil aggregates (30–50 cm) might be attributed to the lower degradability of SOC in this soil layer due to the less exposure to oxygen and microbial activities.

Resistant organic C (ROC) refers to the long-term stable forms of soil C pool with a high radiocarbon age [48]. Hence, it strongly effects the accumulation of SOC [39]. Herein, the continuous accumulation of ROC in SF and SDF top soils (0–30 cm) was mainly related to the high C/N ratio of straw, as well as the abundance of hardly degradable lignin, cellulose, and hemicellulose (Table 3) [49]. Conversely, insignificant differences happened in ROC contents among the different treatments in 30–50 cm due to the shallow straw incorporation in this study [50,51].

Higher DOC contents in SF and to a less extend in SDF than the other treatments might attribute to the direct inputs of C and N via the combined chemical fertilization and straw returning, stimulating the soil microbial functionality (Table 4) [52,53]. Similar observations occurred in Hu et al. [45]. Unlike, a reduction appeared in DOC content of straw amended Jiangxi paddy soils due to the higher biodegradability/consumption of dissolved organic matter by microorganisms [19]. Comparatively, in this study, higher DOC contents happened in deep soil layers (30–50 cm) relative to the surface soil layers (0–30 cm). This can be explained by the effects of hydrology and intensive tillage [19,43]. Particularly, higher precipitation in the early rice season might increase DOC leaching in the experimental site [19]. It is also worth noting that the lower DOC contents of the smaller aggregates might attribute to their less capability/protection against subsurface DOC leaching [19].

### 4.3 Crop yield

The significant effect of co-application of straw returning and chemical fertilization on the crop yields in the study site was in agreement with several recent studies [4,54,55]. Shan et al. [54] suggested that higher crop yields in the straw amended soils primarily attributes to the long-term significant inputs of C and nutrients by straw [54]. In addition, straw returning together with chemical fertilization promotes the microbial diversity and population, that in turn actively contributes to the straw decomposition [4]. This, in particular, happens during the anaerobic rice cultivation. However, straw returning alone might inhibit the crop growth due to the N deficiency brought by the microbial fixation of available N, as well as increasing the C/N ratio [4,54]. Coupled with chemical N fertilization, increases happen in crop productivity due to the application of basal N [4,56]. These explain higher yields of both rice and rapeseed crops in SF than in SDF (Table 5). Besides, long-term *in situ* straw retuning improves the water holding capacity of the soil via higher porosity and less evaporation. The latter regulates the soil temperature and humidity for a better crop growth and a higher yield [55].

## 5 Future research gaps

Despite the successful documentation of the effects of long-term *in situ* straw returning on soil aggregates’ formation, aggregate-associated organic C fractions, and crop yield in the present work, the following points still merit further attention in the future studies.

• As also addressed in some recent works [40,57], the accumulation of different SOC fractions should be investigated in relation to the soil properties, particularly pH, Fe/Al oxides, as well as the available/total contents of major nutrients.
• Future studies should monitor the temporal/spatial changes of EOC contents of the straw amended soils in detail, and characterize the mechanisms undertaking the EOC variations specially in relation to the emissions of greenhouse gases.
• It is essential to examine the effects of different modes/depths of straw returning and tillage practices on the soil OC factions in the study area [44]. In addition, an efficient fertilization scheme can be suggested via returning the crop residues alone or together with chemical fertilization.
• The impact of straw incorporation on crop production should be studied in a longer time period. Han et al. [58] suggested that the highest crop yield happened after 11–15 years of straw returning.
• The rate of straw decomposition, soil aggregation, and the accumulation of different OC fractions merit further investigation in regards to the C/N ratio, microbial/enzymes' activity, and the irrigation regimes.

## 6 Conclusion

The present work successfully examined the efficiency of long-term *in situ* straw returning combined with the conventional fertilization on soil aggregation, aggregate-associated C fractions, and crop yield in Chaohu Lake Region, Anhui Province, East China. Although large macro-aggregates (>2 mm) were dominant in all treatments, their highest contents happened in SF and SDF soils. Further, straw returning significantly increased the aggregates' associated organic C contents with higher accumulations in topsoil layers (0–30 cm). In particular, the highest TOC contents took place in macro-aggregates of straw amended top soils. Similar observations occurred for the contents of EOC, DOC, and ROC. It is also worth noting that the highest TOC, EOC, and ROC contents appeared in 0.25–2 mm aggregates, while > 2 mm aggregates had the highest contents of DOC. Finally, the highest seasonal yields of rapeseed and rice happened in SF soils, whereas no significant difference was found in the annual productions of both crops in F and SDF treatments. Future works should further investigate the spatiotemporal changes of soil OC fractions in relations to the rate and the mode of straw returning, C/N ratio, microbial/enzymes’ activity, irrigation regimes, and different soil properties.

## CRediT authorship contribution statement

**Jiaren Liu:** Writing – original draft, Software, Methodology, Investigation, Formal analysis, Data curation. **Xuehai Wang:** Writing – original draft, Methodology, Formal analysis, Data curation. **Hu Zhang:** Methodology, Data curation. **Yifei Lu:** Methodology, Funding acquisition, Data curation. **Yusef Kianpoor Kalkhajeh:** Writing – review & editing, Supervision, Conceptualization. **Hongxiang Hu:** Visualization, Investigation, Conceptualization. **Jieying Huang:** Writing – review & editing, Supervision, Resources, Methodology, Funding acquisition, Conceptualization.

## Declaration of competing interest

The authors declare that they have no known competing financial interests or personal relationships that could have appeared to influence the work reported in this paper.
